# miR-182-5p attenuates *Schistosoma japonicum*-induced hepatic fibrosis by targeting tristetraprolin: miR-182-5p attenuates hepatic fibrosis

**DOI:** 10.3724/abbs.2022130

**Published:** 2022-09-22

**Authors:** Xuejun Zhao, Zijie Xia, Ziang Wang, Mengsi Zhou, Xuebing Qiu, Cheng Wang, Tian Xu, Qian Fang, Zhenping Ming, Huifen Dong

**Affiliations:** 1 Hubei Province Key Laboratory of Allergy and Immunology School of Basic Medical Sciences Wuhan University Wuhan 430071 China; 2Department of Medical Parasitology School of Basic Medical Sciences Wuhan University Wuhan 430071 China; 3 Kingstar Global Wuhan 430070 China

**Keywords:** *Schistosoma japonicum*, microRNA, liver fibrosis, miR-182-5p, tristetraprolin

## Abstract

Egg granuloma formation in the liver is the main pathological lesion caused by *Schistosoma japonicum* infection, which generally results in liver fibrosis and may lead to death in advanced patients. MicroRNAs (miRNAs) regulate the process of liver fibrosis, but the putative function of miRNAs in liver fibrosis induced by *S*. *japonicum* infection is largely unclear. Here, we detect a new miRNA, miR-182-5p, which shows significantly decreased expression in mouse livers after stimulation by soluble egg antigen (SEA) of *S*. *japonicum* or *S*. *japonicum* infection. Knockdown or overexpression of miR-182-5p *in vitro* causes the increased or decreased expression of tristetraprolin (TTP), an important immunosuppressive protein in the process of liver fibrosis. Furthermore, knockdown of miR-182-5p *in vivo* upregulates TTP expression and significantly alleviates *S*. *japonicum*-induced hepatic fibrosis. Our data demonstrate that downregulation of miR-182-5p increases the expression of TTP in mouse livers following schistosome infection, which leads to destabilization of inflammatory factor mRNAs and attenuates liver fibrosis. Our results uncover fine-tuning of liver inflammatory reactions related to liver fibrosis caused by *S*. *japonicum* infection and provide new insights into the regulation of schistosomiasis-induced hepatic fibrosis.

## Introduction

Schistosomiasis is one of the serious tropical diseases caused by three main species of schistosomes: *Schistosoma mansoni*, *S*. *haematobium*, and *S*. *japonicum* [1]. It affects over 700 million people living in endemic areas, with at least 240 million patients [2]. During *S*. *mansoni* and *S*. *japonicum* infection, eggs are deposited in the liver, which induces the host immune response and eventually may lead to chronic liver injury and induce liver fibrosis [ 1, 3] .

MicroRNAs (miRNAs) are a group of endogenous, highly conserved noncoding RNAs that are involved in many biological processes via their role in mRNA degradation or translational repression [ 4‒ 6] . A large number of studies have suggested that miRNAs act as potential therapeutic targets in liver diseases [ 7, 8] . Several miRNAs have been identified as important players in the progression of hepatic fibrosis caused by schistosome infection. miR-454 inhibits the activation of HSCs by directly targeting Smad4 [9]. Inhibition of miR-21 using highly hepatic tropic adeno-associated virus serotype 8 (rAAV8) protects mice against lethal schistosome infection through attenuation of hepatic fibrosis *in vivo* [10]. Our previous study also showed that inhibition of miR-27b attenuates schistosome-induced hepatic fibrosis [11]. However, the mechanisms underlying the function of miRNAs following *S*. *japonicum* infection are largely unclear.

During chronic liver injury, hepatocytes undergoing cellular injury and death produce a variety of inflammatory cytokines, such as tumor necrosis factor (TNFα), IL-6 (interleukin 6), and C-X-C motif chemokine ligand 1 (CXCL1), which play major roles in the pathogenesis of liver fibrosis [ 12‒ 14] . Intriguingly, a recent study reported that tristetraprolin (TTP, also known as ZFP36) ameliorates hepatic injury by regulating the mRNA stabilization of CCL2 and CCL5 *in vivo* [15]. TTP is known as an important immunosuppressive protein that controls the mRNA stability of several inflammatory factors, such as IL-6, interleukin 8 (IL-8), and CCL2, by recognizing the AU-rich elements (AREs) within the 3′UTRs of these mRNAs [16]. Furthermore, it has been reported that overexpression of TTP attenuates liver fibrosis by degrading MMP-2 and TNFα [17].

In this study, we found that the expression of miR-182-5p was downregulated in hepatocytes following *S*. *japonicum* infection and soluble egg antigen (SEA) stimulation. Knockdown of miR-182-5p expression notably increased the expression of TTP at the mRNA and protein levels *in vitro*. Overexpression of miR-182-5p decreased the mRNA and protein levels of TTP. Moreover, *in vivo* knockdown of miR-182-5p in mice upregulated the expression of TTP and attenuated the development of schistosomiasis-related hepatic fibrosis, which may provide a new treatment strategy for liver fibrosis caused by *S*. *japonicum* infection.

## Materials and Methods

### Cell culture and treatments

HL7702 and AML12 were obtained from the American Type Culture Collection, U937 was received from Dr HB Shu (Wuhan University, Wuhan, China) originally obtained from ATCC. Cells were cultured in DMEM supplemented with 10% fetal bovine serum (Gibco, Carlsbad, USA). Isolation of primary hepatocytes from female mice (BALB/C, 6 weeks old) was performed as described previously [18]. SEA was obtained from purified schistosome eggs from the livers of infected mice, as previously described [19].

SEA was used to induce cell lines at the following concentrations: 30 μg/mL for HL7702, AML12, and U937 cells and 60 μg/mL for primary hepatocytes. Cells were transfected with miR-182-5p mimics to increase miR-182-5p (50 and 100 nM; RIBOBO, Guangzhou, China), miR-182-5p inhibitor to knockdown miR-182-5p (50 and 100 nM; RIBOBO). Cells were treated with IFNγ (10 ng/mL; Sigma, St Louis, USA). Cells were transfected with Dicer siRNA and Drosha siRNA (50 nM; GenePharma, Suzhou, China). The sequences are listed in Supplementary Table S1.

### Ethics statement

Animal experiment protocols were approved by the Animal Care and Use Committee of Wuhan University, and all experiments were performed following the guidelines and regulations of Wuhan University. All surgeries were performed under ketamine (100 mg/kg body weight, *i*. *p*.) and xylazine (10 mg/kg body weight, *i*. *p*.) anesthesia, and all efforts were made to minimize the suffering of the animals.

### Mice and parasitic infection

Six-week-old female BALB/C mice (weighing 18–20 g) were purchased from China Three Gorges University (Yichang, China) and maintained in a specific pathogen-free environment. *Oncomelania hupensis* snails were provided by the Jiangsu Institute of Parasitic Disease (Wuxi, China). Anesthetized mice were percutaneously infected with 16 cercariae of *S*. *japonicum*.

### Hydrodynamic tail-vein injection

According to our previous study [11], pEGFPC1-miR-182-5p-sponge was generated and suspended in PBS and, in less than 7 seconds, injected into the lateral tail veins of mice (0.1 mL/g body weight) infected with parasites for 6 weeks. Hydrodynamic injection was performed once every 3 days. Livers were collected two weeks after the hydrodynamic transfection.

### Generation of *TTP*-knockdown and overexpression cells

According to our previous study [15], HL7702 cells were seeded into a well and transfected with indicated lentivirus according to the instructions. Infected cells were selected using 1 μg/mL puromycin (MACKLIN, Shanghai, China) for 1 week. For the TTP overexpression experiments, cells in 6-well plates were transfected with 2 μg PHAGE-TTP plasmid (PAGHE, #118692; Addgene, Watertown, USA). The shTTP, shCtrl, and PHAGE-TTP (TTP-OE) sequences are listed in Supplementary Table S1.

### RNA extraction and real-time PCR

Total RNA was extracted using TRIzol reagent (Takara, Dalian, China) according to the manufacturer’s protocol. The expression levels of miR-182-5p, *Col1α1*, *Col3α1*, *α-SMA*, *TTP*, *IL-6*, *IL-8*, and *TNFα* were detected by real-time PCR using the SYBR Green Master Mix Kit (Vazyme, Nanjing, China). *U6*, *ACTIN* *or GAPDH* was used as an endogenous control, and the fold-change was calculated by the 2 ^−ΔΔCt^ method. The sequences of the primers are shown in Supplementary Table S1.

### Western blot analysis

Proteins were extracted with RIPA lysis buffer (Servicebio, Wuhan, China), quantified by the BCA method (Biosharp, Beijing, China), loaded in SDS-PAGE gels to separate proteins and transferred to polyvinylidene fluoride membranes. After being blocked with 5% non-fat milk in TBST for 1 h, membranes were incubated with primary antibodies at 4°C overnight. After wash with TBST for three times, membranes were incubated with HRP-conjugated secondary antibodies for 1 h at room temperature. Antibodies against TTP (1:1000; Millipore, Billerica, USA), Tubulin (1:1000; Proteintech, Rosemont, USA), GAPDH (1:10000; Proteintech), IL-8 (1:1000; Proteintech), TNFα (1:1000; Proteintech), and IL-6 (1:500; ABclonal, Wuhan, China) were used as the primary antibodies.

### mRNA stability assay

HL7702 cells were transfected with miR-182-5p mimics (50 nM). Then, the cells were exposed to actinomycin D (5 ng/mL; MCE, New Jersey, USA ) and collected at 1 and 2 h after actinomycin D treatment. Total RNA was isolated and subsequently subjected to real-time PCR to quantify the relative abundance of TTP mRNA (relative to 0 h).

### Luciferase reporter assay

HL7702 cells were seeded into a 24-well plate before transfection. Full-length TTP 3′UTR and truncated TTP 3′UTR genes were amplified by PCR and then cloned into the luciferase vector, pMIR-GLO, to generate the reporter plasmids. The pMIR-GLO reporter vectors containing the truncated TTP 3′UTR and the mutated 3′UTR or full-length TTP 3′UTR and mutated full-length TTP 3′UTR fragment were transfected into cells using 1 μL of Lipofectamine 2000 transfection reagent (Invitrogen, Carlsbad, USA) following the manufacturer’s instructions. After 24 h of transfection, the cells were collected into reporter lysis buffer (Promega, Madison, USA), and luciferase activity was detected using a luciferase assay system (Promega). The firefly luciferase activities were normalized to the Renilla luciferase activities. The primers for constructing the mutated 3′UTR fragment are listed in Supplementary Table S1.

### Histology and immunohistochemistry

Liver samples were fixed with 4% paraformaldehyde and embedded in paraffin. Sectioned tissues were stained with Mayer’s H&E to measure the size of hepatic granulomas using a calibrated measuring eyepiece. In addition, tissues were stained with Masson’s trichrome to determine the extent of fibrosis. Liver sections (4 μm) were deparaffinized in xylene and hydrated using an ethanol-deionized water series. Ethylene Diamine Tetraacetic Acid (EDTA) repair solution was used for antigen repair by microwave. The sections were incubated with 3% H _2_O _2_ for 10 min at room temperature, and then incubated with primary antibodies, including anti-α-SMA (1:1000; Proteintech) and anti-COL1A1 (1:5000; Proteintech) antibodies. Then, the sections were incubated with horseradish peroxidase-labeled secondary antibody at room temperature for 1 h. The sections were developed with DAB reagent solution, and nuclei were stained with hematoxylin solution. Then, the sections were dehydrated, transparent, and sealed with neutral resin, followed by immunohistochemical examination under a microscope.

### Statistical analysis

All data are presented as the means±SD of three experiments. Analysis was performed using Student’s *t*-test or ANOVA when appropriate. *P* values of <0.05 were considered statistically significant.

## Results

### SEA stimulation and *S*. *japonicum* infection decreases the expression of miR-182-5p *in vitro* and *in vivo*

**Figure 1:**
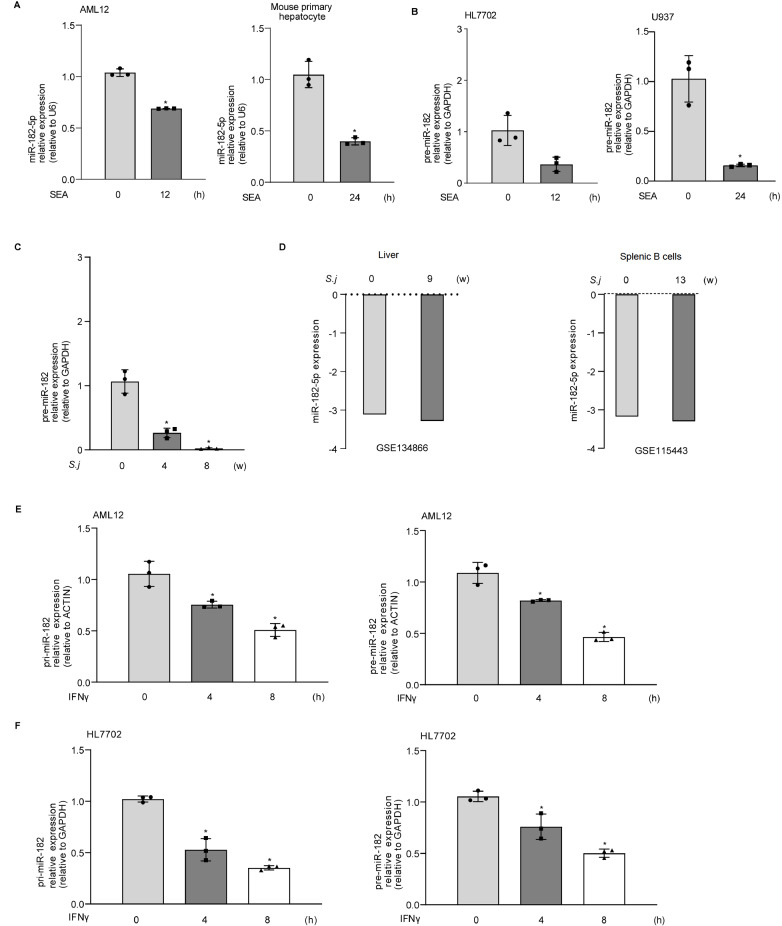
Figure 1 The expression of miR-182-5p following SEA, IFNγ stimulation and *S*. *japonicum* infection *in vitro* and in *vivo* (A) The expression of miR-182-5p in AML12 and primary hepatocytes exposed to SEA (AML12, 30 μg/mL, mouse primary hepatocytes, 60 μg/mL) for 12 and 24 h. (B) The expression of pre-miR-182 in HL7702 and U937 cells after exposure to SEA (30 μg/mL) for 12 and 24 h. (C) The expression of pre-miR-182 in mouse livers 4 and 8 weeks after S. japonicum infection. (D) The expression of miR-182-5p in mouse livers (GSE134866) and splenic B cells (GSE115443) 9 and 13 weeks after S. japonicum infection. (E) pri-miR-182 and pre-miR-182 expression in AML12 cells exposed to IFNγ for up to 8 h. (F) pri-miR-182 and pre-miR-182 expression in HL7702 cells exposed to IFNγ for up to 8 h. * P<0.05.

We first explored miR-182-5p expression in mouse hepatocytes (AML12), mouse primary hepatocytes, human hepatocytes (HL7702), and human macrophages (U937) in response to SEA induction. The expressions of pre-miR-182 and miR-182-5p were decreased in AML12, mouse primary hepatocytes, HL7702, and U937 cells in response to SEA stimulation ( Figure 1A,B). Consistent with the *in vitro* data, a significant decrease in pre-miR-182 expression was detected in the livers of mice infected with *S*. *japonicum* for 4 and 8 weeks ( Figure 1C). Previously published genome-wide expression profiling data (GSE134866) showed that downregulation of miR-182-5p was detected in the livers of mice infected with *S*. *japonicum* for 9 weeks ( Figure 1D). In addition, publicly available mouse transcriptome datasets (GSE115443) supported the downregulation of miR-182-5p in splenic B cells of C57BL/6 mice in the late phase of *S*. *japonicum* infection ( Figure 1D).

IFNγ, a type II interferon with important immunomodulatory properties, triggers the transactivation of many inflammatory-related genes, contributing to the pathogenesis of schistosome-induced hepatic fibrosis [20]. Therefore, we assessed the expressions of pre-miR-182 and pri-miR-182 in cultured hepatocytes (HL7702 and AML12) in response to IFNγ stimulation. Decreased pre-miR-182 and pri-miR-182 levels were detected in HL7702 and AML12 cells following IFNγ treatment for 4 and 8 h ( Figure 1E,F).

### miR-182-5p represses the expression of TTP in hepatocytes

**Figure 2:**
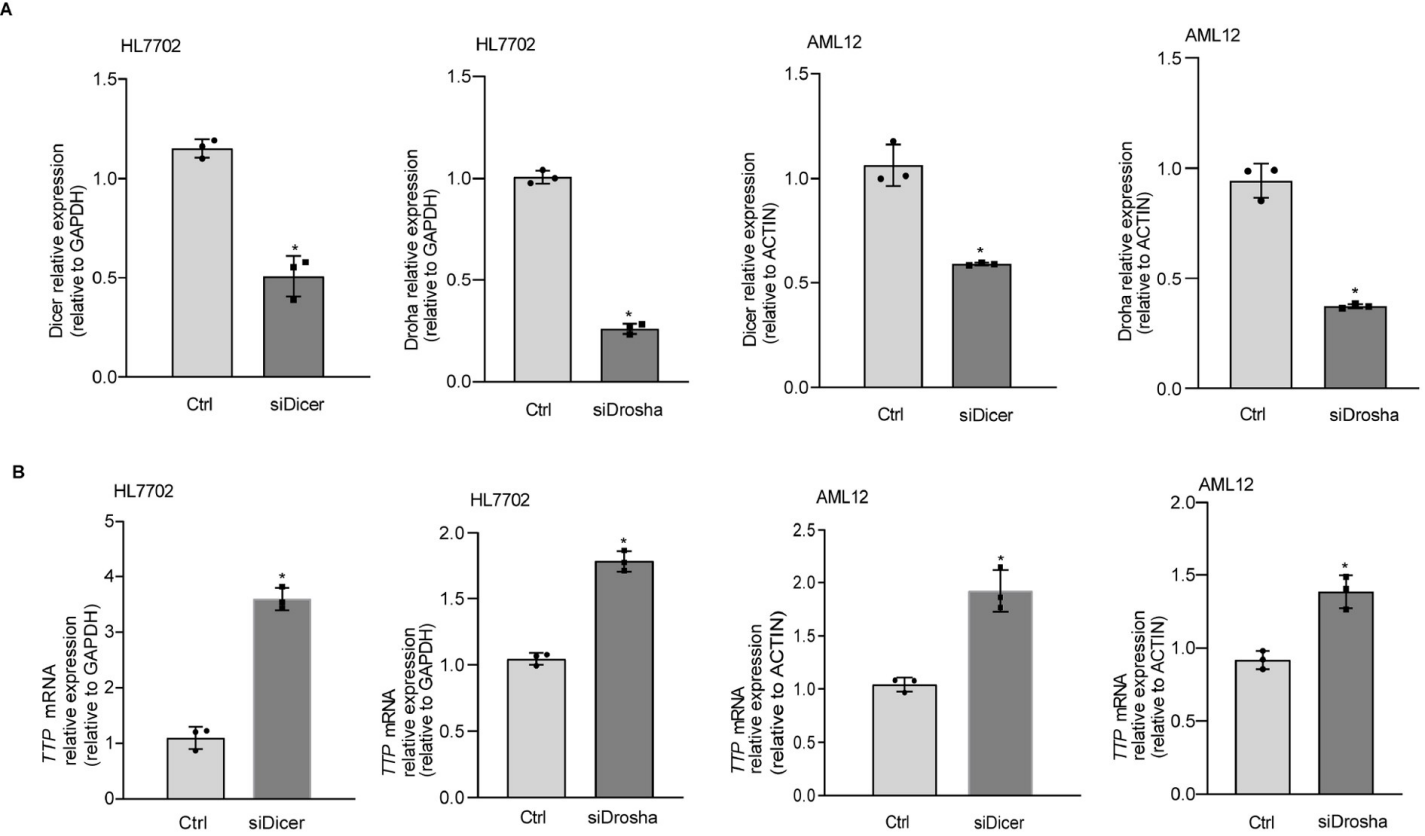
Figure 2 TTP expression is inhibited in cells transfected with siDicer and siDrosha *in vitro* (A) The efficiency of siRNA transection. (B) The mRNA level of TTP in cells transfected with siDicer and siDrosha. * P<0.05.

To determine whether miRNAs play roles in the regulation of TTP expression, we designed siRNAs to target regions of Dicer and Drosha to knock down Dicer and Drosha. The efficiency of siRNA for Dicer and Drosha was confirmed in HL7702 and AML12 cells ( Figure 2A). Compared with the control cells, the mRNA level of *TTP* was significantly increased in cells transfected with siDicer and siDrosha ( Figure 2B).

**Figure 3:**
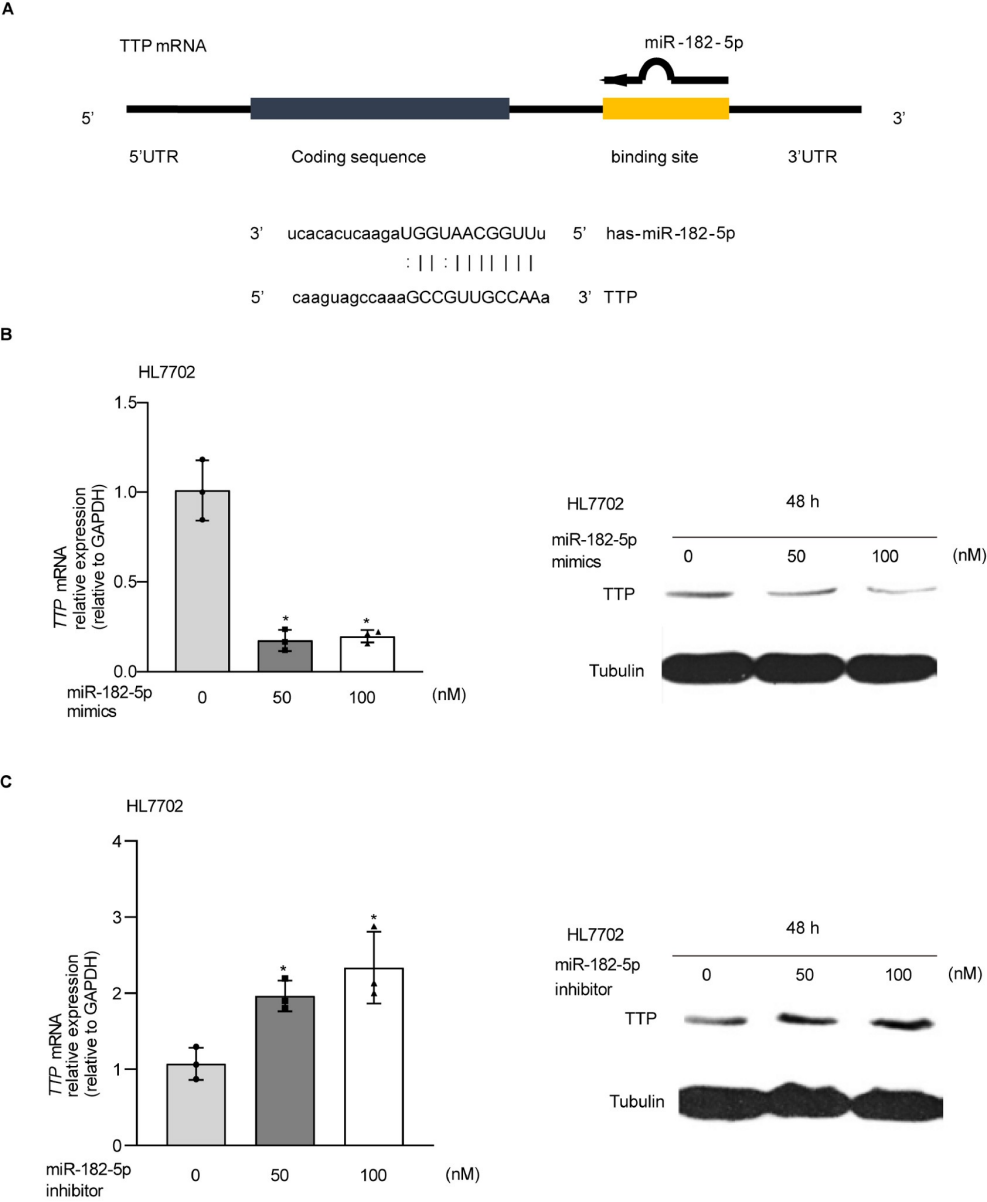
Figure 3 miR-182-5p regulates TTP expression in hepatocytes (A) The putative binding sites for miR-182-5p in TTP. (B) TTP mRNA and protein levels in cells transfected with miR-182-5p mimic. (C) TTP mRNA and protein levels in cells transfected with miR-182-5p inhibitor. * P<0.05.

**Figure 4:**
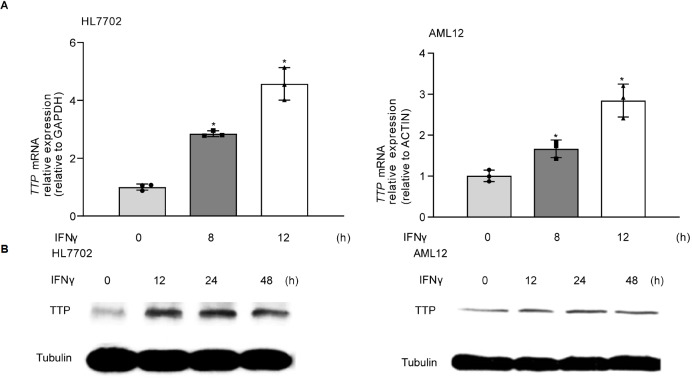
Figure 4 IFNγ increases TTP expression *in vitro* (A) AML12 and HL7702 cells were exposed to IFNγ for up to 12 h, followed by real-time PCR. (B) AML12 and HL7702 cells were exposed to IFNγ for up to 48 h, followed by western blot analysis. * P<0.05.

Using miRBase ( http://www.mirbase.org/), we found that TTP had putative binding sites for miR-182-5p ( Figure 3A). Considering that there are several putative binding sites for miR-182-5p in the TTP 3′UTR, we tested whether overexpression or inhibition of miR-182-5p regulates the expression of TTP at the mRNA and protein levels. Overexpression of miR-182-5p significantly decreased the mRNA and protein levels of TTP in HL7702 cells transfected with miR-182-5p mimics ( Figure 3B). Meanwhile, the miR-182-5p inhibitor increased the expression of TTP in HL7702 cells ( Figure 3C). Subsequently, we investigated TTP expression in HL7702 and AML12 cells following IFNγ stimulation by real-time PCR and western blot analysis. The expression of TTP was significantly increased in cells following IFNγ treatment for 8 and 48 h ( Figure 4).

### miR-182-5p regulates TTP mRNA in a 3′UTR-dependent manner

**Figure 5:**
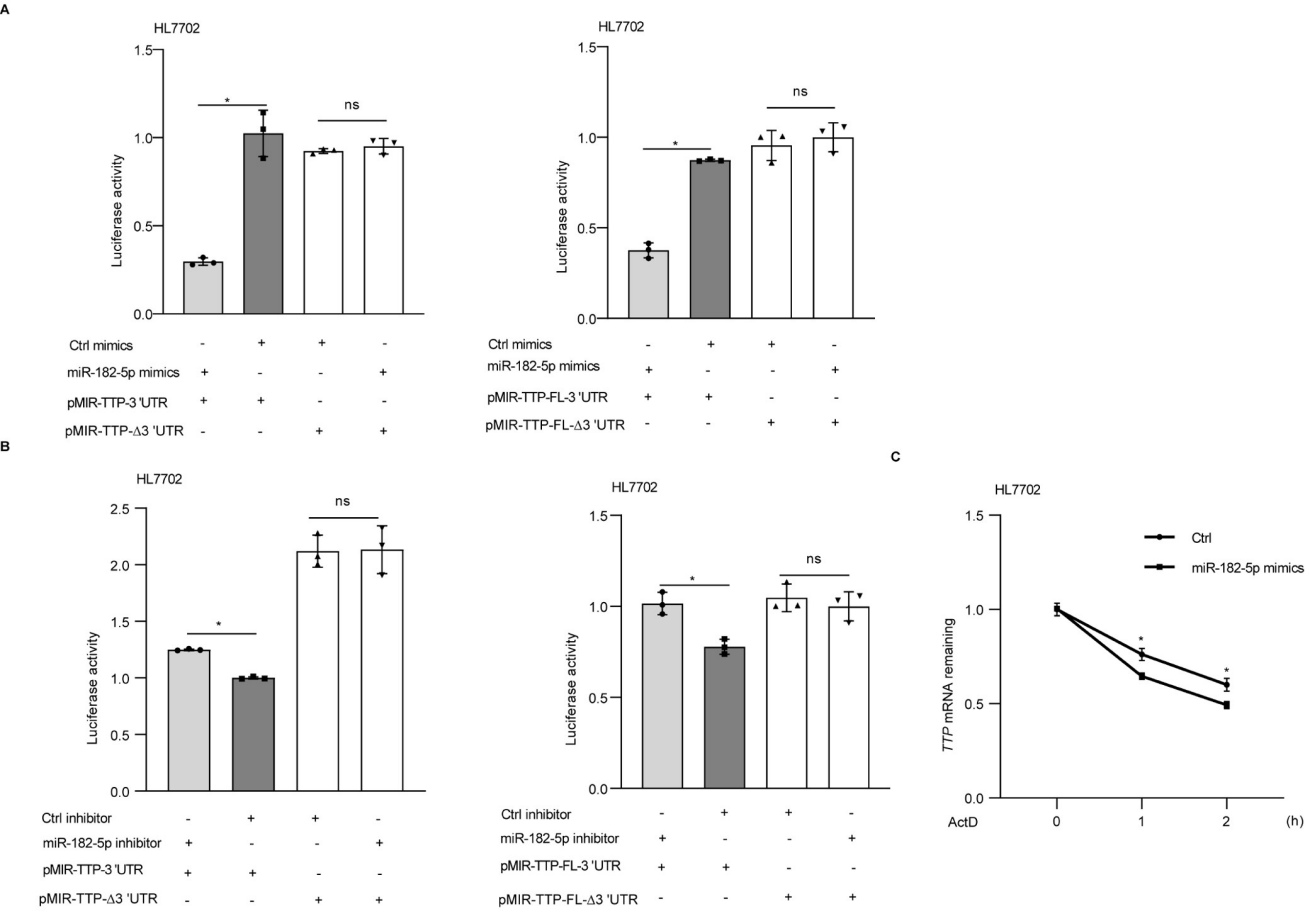
Figure 5 miR-182-5p regulates *TTP* mRNA in a 3′UTR-dependent manner (A) HL7702 cells were cotransfected with pMIR-TTP-3′UTR, pMIR-TTP-∆3′UTR, pMIR-TTP-FL-3′UTR or pMIR-TTP-FL-∆3′UTR together with miR-182-5p mimics. (B) HL7702 cells were cotransfected with pMIR-TTP-3′UTR, pMIR-TTP-∆3′UTR, pMIR-TTP-FL-3′UTR or pMIR-TTP-FL-∆3′UTR together with miR-182-5p inhibitor. (C) The stability of TTP mRNAs was calculated in HL7702 cells transiently transfected with miR-182-5p mimics. Actinomycin D (Act D) was then added, and the cells were collected for real-time PCR. The stability of mRNAs is presented as the relative amount of mRNA to the control. * P<0.05. ns, no significance.

To elucidate whether miR-182-5p can bind to the TTP 3′UTR, thus regulating TTP, we used four constructs containing luciferase cDNA: truncated TTP 3′UTR (pMIR-TTP-3′UTR), truncated TTP 3′UTR with mutated binding sites (pMIR-TTP-∆3′UTR), full-length TTP 3′UTR (pMIR-TTP-FL-3′UTR), and full-length TTP 3′UTR with mutated binding sites (pMIR-TTP-FL-∆3′UTR). HL7702 cells were cotransfected with pMIR-TTP-3′UTR, pMIR-TTP-∆3′UTR, pMIR-TTP-FL-3′UTR, or pMIR-TTP-FL-∆3′UTR together with the miR-182-5p mimics. miR-182-5p mimic transfection showed a significant decrease in luciferase activity in cells transfected with the pMIR-TTP-3′UTR or pMIR-TTP-FL-3′UTR ( Figure 5A). In cells transfected with the 3′UTR mutant constructs , the associated luciferase activity was not changed ( Figure 5A). Meanwhile, miR-182-5p inhibitor transfection showed a significant increase in luciferase activity in cells transfected with pMIR-TTP-3′UTR or pMIR-TTP-FL-3′UTR ( Figure 5B). In cells transfected with the construct containing the mutation of the FL-3′UTR, the associated luciferase activity was not changed ( Figure 5B). Furthermore, *TTP* mRNA stability was measured in HL7702 cells transfected with miR-182-5p mimics. As shown in Figure 5C, HL7702 cells transfected with miR-182-5p mimics displayed a significant decrease in *TTP* mRNA stability.

### miR-182-5p influences the expression of fibrosis-related cytokines

**Figure 6:**
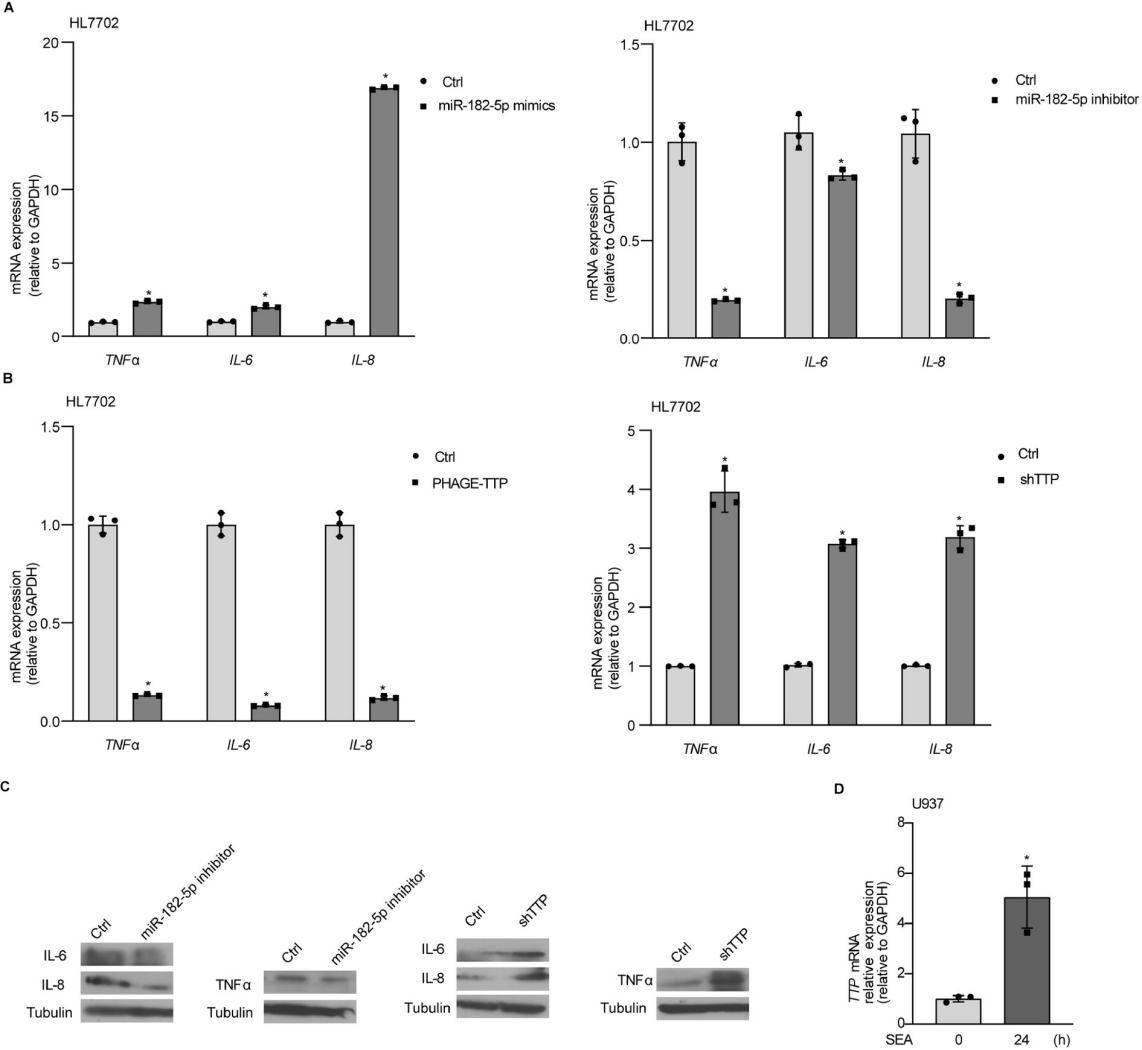
Figure 6 miR-182-5p influences the expression of fibrosis-related cytokines (A) The expressions of selected fibrosis-related cytokines in HL7702 cells transfected with miR-182-5p mimics or miR-182-5p inhibitor. (B) Real-time PCR for fibrosis-related cytokine mRNA in HL7702 cells transiently transfected with PHAGE-TTP and in stable TTP-knockdown HL7702 cells. (C) The protein levels of IL-6, IL-8, and TNFα were detected in HL7702 cells transiently transfected with miR-182-5p inhibitor and in stable TTP-knockdown HL7702 cells. (D) The expression level of TTP in U937 cells after exposure to SEA (30 μg/mL) for 24 h. * P<0.05.

To clarify the potential role of miR-182-5p in regulating the expression of fibrosis-related cytokines, we investigated the effects of downregulation and overexpression of miR-182-5p on the expressions of several fibrosis-related cytokines, including TNFα, IL-6, and IL-8. Overexpression of miR-182-5p increased the mRNA levels of *TNFα*, *IL-6*, and *IL-8*. Knockdown of miR-182-5p decreased the mRNA levels of *TNFα*, *IL-6* and *IL-8* ( Figure 6A). Meanwhile, overexpression of TTP decreased the mRNA levels of *TNFα*, *IL-6*, and *IL-8*. Knockdown of TTP increased the mRNA levels of *TNFα*, *IL-6*, and *IL-8* ( Figure 6B). In addition, we also measured the protein levels of TNFα, IL-6, and IL-8 in cells transfected with miR-182-5p inhibitor or TTP shRNA. Knockdown of miR-182-5p decreased the protein levels of TNFα, IL-6, and IL-8. TTP depletion increased the protein levels of TNFα, IL-6, and IL-8 ( Figure 6C). Increase of TTP levels were also observed in U937 cells following SEA treatment for 24 h ( Figure 6D).

### miR-182-5p knockdown attenuates schistosomiasis-induced hepatic fibrosis

**Figure 7:**
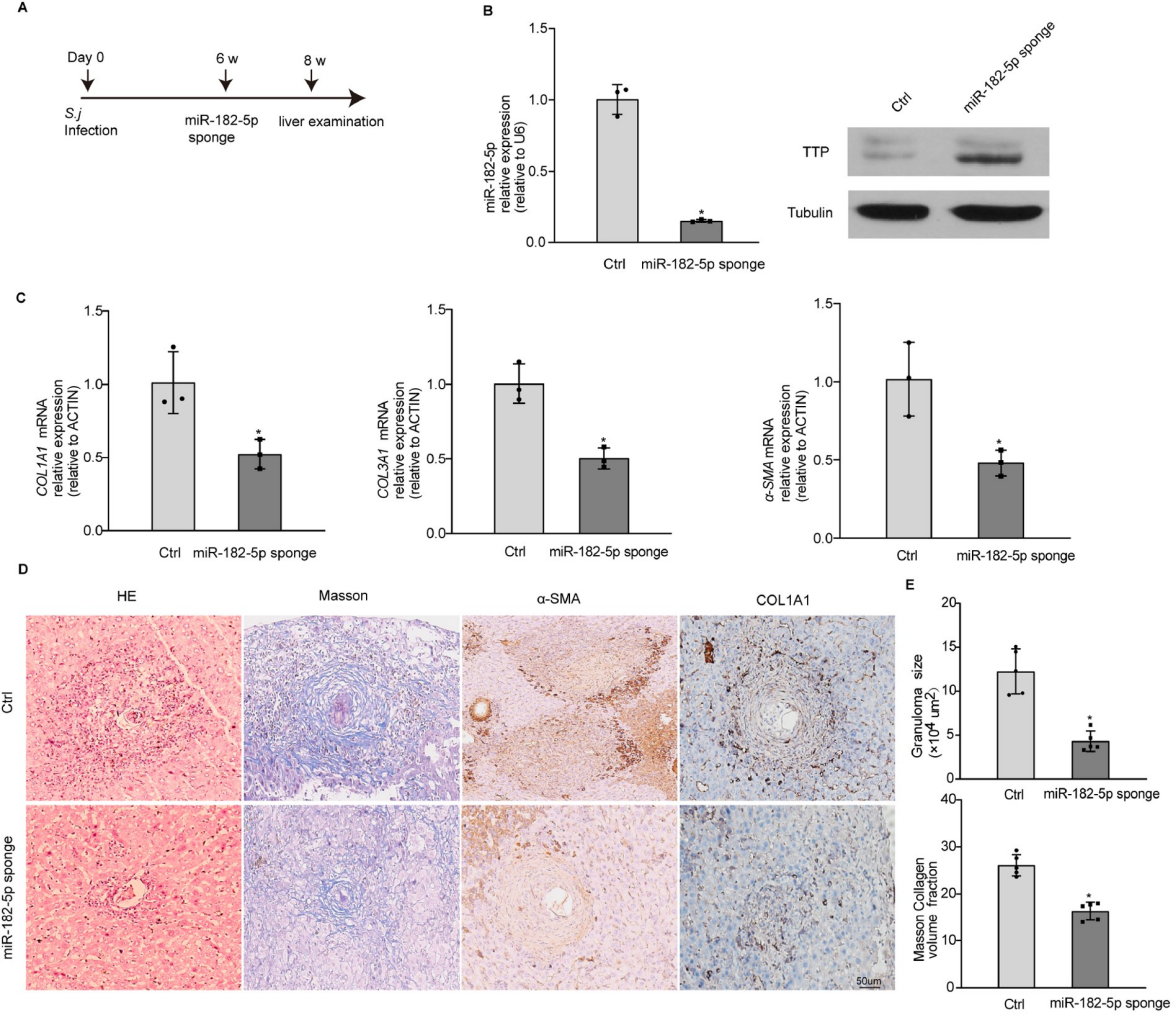
Figure 7 miR-182-5p knockdown attenuates schistosomiasis-induced hepatic fibrosis (A) Schematic overview of parasite infection and administration of plasmid vectors and samples collected. (B) Real-time PCR analysis of the levels of mature miR-182-5p and western blot analysis of TTP expression in liver tissues. (C) Real-time PCR analysis of the levels of Col1α1, Col3α1, and α-SMA mRNA in liver tissues. (D) Masson’s trichrome staining and H&E staining of liver sections. Liver sections were immunohistochemically stained for α-SMA and COL1A1. Scale bar: 50 μm. (E) Granuloma size was measured by H&E staining and ECM deposits were detected by Masson’s trichrome staining in liver sections. * P<0.05.

To investigate the role of miR-182-5p in schistosomiasis-induced liver fibrosis *in vivo*. Mice were infected with *S*. *japonicum* cercariae and subsequently injected with hydrodynamic miR-182-5p sponge vectors or control vectors through the tail vein at 42 days postinfection ( Figure 7A). The expression of miR-182-5p was significantly decreased in mice that received the miR-182-5p sponge compared with the controls ( Figure 7B). The protein level of TTP was increased in the miR-182-5p sponge-treated group compared with the control group ( Figure 7B). The expressions of *COL1A1*, *COL3A1*, and *α-SMA* were significantly reduced in the mice treated with a miR-182-5p sponge ( Figure 7C). The protein levels of α-SMA and COL1A1 were decreased in the mice treated with the miR-182-5p sponge ( Figure 7D). The size of egg granulomas was markedly reduced in the miR-182-5p sponge-treated mice, as visualized by H&E staining ( Figure 7D,E). In addition, mice injected with the miR-182-5p sponge displayed a significant reduction in extracellular matrix (ECM) deposits, as demonstrated by Masson′s trichrome staining ( Figure 7D,E). Taken together, our data show that inhibition of miR-182-5p can attenuate schistosomiasis-related hepatic fibrosis.

## Discussion

In this study, we revealed the downregulation of miR-182-5p in hepatocytes in response to SEA stimulation and *S*. *japonicum* infection. Using the miR-182-5p sponge and hydrodynamic injection, we found that inhibition of miR-182-5p protects against the effects of schistosome infection by alleviating hepatic fibrosis *in vivo*. Mechanistically, miR-182-5p regulates the expression of TTP mRNA by interacting with its 3′UTR in hepatocytes, which affects the process of liver fibrosis during *S*. *japonicum* infection.

Hepatic fibrosis is the pathological response that may cause chronic liver diseases. At present, there is still no safe and effective treatment strategy for hepatic fibrosis, including schistosome-induced hepatic fibrosis [ 21, 22] . miRNAs have been reported to play important roles in the development of hepatic fibrosis by modulating gene expression at the posttranscriptional level. There is considerable evidence demonstrating that targeting dysregulated miRNAs by using gene delivery systems is a promising therapeutic intervention for hepatic fibrosis [23]. A previous study reported that sustained elevation of miR-203-3p in liver tissues protected mice against schistosome infection [24]. Inhibition of miR-351 protected hosts from the lethal effects of schistosome infection by alleviating hepatic fibrosis [25]. It has been reported that miR-182-5p is the most highly expressed miRNA in alcoholic liver disease (ALD) and that it is correlated with disease severity and liver injury [26]. miR-182-5p significantly increases lipid accumulation in ALD by targeting FOXO1 [27]. Interestingly, Dolganiuc *et al*. [28] reported that miR-182 was significantly downregulated in mice fed with a Lieber-de Carli alcohol diet compared to controls. The role of miR-182-5p expression in liver diseases remains controversial, and the molecular mechanism underlying the function of miR-182-5p remains to be elucidated. Our results show that SEA stimulation and *S*. *japonicum* infection result in downregulation of miR-182-5p in the mouse liver. Considering the important role of IFNγ in the pathogenesis of schistosome-induced hepatic fibrosis [20], we also investigated the effect of IFNγ on miR-182 expression, and our data indicated that IFNγ significantly reduced miR-182 expression in hepatocytes and knockdown of miR-182-5p decreased the expression of fibrosis-related cytokines. Importantly, our results show that *S*. *japonicum*-induced hepatic fibrosis in mice treated with miR-182-5p sponge vectors is significantly reduced, which suggests that targeting miR-182-5p may be a useful therapy not only for schistosomiasis infection-induced hepatic fibrosis but also for other types of hepatic fibrosis.

TTP is an RNA-binding protein (RBP) that plays an important role in the posttranscriptional control of immune inflammatory responses. It has been reported that TTP attenuates inflammasome-driven fibrogenesis by serving as a negative regulator of NLRP3 [ 29, 30] . Patial *et al*. [31] TTP has a protective effect against several models of immune and inflammatory disease using the TTP-overexpressing mouse model. It has been reported that TTP overexpression results in autophagy inactivation by decaying *ATG16L1* mRNA, which posttranscriptionally regulates HSC survival and death in liver fibrosis [32]. In this study, we found that miR-182-5p regulated TTP expression by binding to the TTP 3′UTR in hepatocytes, and overexpression of TTP decreased the mRNA levels of *TNFα*, *IL-6*, and *IL-8*. Meanwhile, mice receiving the miR-182-5p-sponge displayed a significant increase in the expression of TTP. The expression of TTP was increased in U937 cells after SEA treatment. TTP can act as a key regulator of the inflammatory response, especially in the fine-tuning of proinflammatory cytokines and chemokines in the liver [33]. A significant increase in the number of F4/80 ^lo^CD11b ^hi^ infiltrating macrophages has been reported in *TTP*-knockdown mice [15]. Our data imply that miR-182-5p regulates the expression of several inflammatory-related genes by targeting *TTP*, which alleviates hepatic fibrosis.

In summary, our data demonstrate that miR-182-5p regulates the expression of TTP to attenuate schistosome-induced hepatic fibrosis, which provides new insights into the pathogenesis of schistosomiasis-induced hepatic fibrosis. In addition, manipulation of miR-182-5p might be a useful strategy not only for treating schistosome-induced hepatic fibrosis but also for curing hepatic fibrosis in general.

## Supporting information

TABLES_S1

## Supplementary Data

Supplementary data is available at *Acta Biochimica et Biophysica Sinica* online.
