# Supplementary material for: miR-182-5p attenuates Schistosoma japonicum-induced hepatic fibrosis by targeting tristetraprolin : miR-182-5p attenuates hepatic fibrosis
Source: Acta Biochim Biophys Sin (Shanghai). 2022 Sep 22;54(10):1421–30. doi: 10.3724/abbs.2022130 (PMC9828319; doi:10.3724/abbs.2022130)
Supplement: TABLES_S1 [file TABLES_S1.pdf]

Supplementary Table S1. The sequences of shRNA and primers used in this study

Gene	Forward (5′→3′)	Reverse (5′→3′)
shCtrl	TTCTCCGAACGTGTCACGTT	
TTP shRNA	CCAGAGCATCAGCTTCTCGAGAAGCT	
h-pri-miR-182	CCAACTATGGGGCGAGGACT	GGAGAACAGCAGGTCCAGCA
h-pre-miR-182	TTTGGCAATGGTAGAACTCACA	GCCCCATAGTTGGCAAGTC
h-*GAPDH*	ACCATCTTCCAGGAGCGAGATC	TGATGACCCTTTTGGCTCCCC
h-Dicer	CAAGTGTCAGCTGTCAGAACTC	CAATCCACCACAATCTCACATG
h-Drosha	TAGGCTGTGGGAAAGGACCAAG	GTTCGATGAACCGCTTCTGATG
h-*TTP*	TCGGGACCCTGGAGCCTGAG	AGCCAGCGGTGCGAAGCC
m-pri-miR-182	TTTTGGCAATGGTAGAACTCAC	CAGGGAAACATTAAGGGTCA
m-pre-miR-182	TTTGGCAATGGTAGAACTCAC	GGCAAGTCTAGAACCACCG
m-Dicer	ACAGCGGGAACGAGACGA	AAAGGACCCATTGGTGAGGA
m-Drosha	ACCAACCCTGGGACGAAAC	AAAGGACCCATTGGTGAGGA
m-*ACTIN*	TGGTGGGAATGGGTCAGAA	TCTCCATGTCGTCCCAGTTG
m-*GAPDH*	ATGGTGAAGGTCGGTGTGAA	CGCTCCTGGAGATGGTGAT
m-*Col1A1*	GCACGAGTCA CACCGGAAC	CCAATGTCCAAGGGAGCCAC
m-*Col3A1*	TGGTCCTCAGGGTGTAAAGG	GTCCAGCATCACCTTTTGGT
h-TTP-3'UTR	ACGCTGAGCTCTTTAAGGGAGGCAATGAACC	ATGCGAAGCTTAGGGAAGCAGACGACCCAAT
h-TTP-FL-3'UTR	ATCATGAGCTCCACGT-<br>CTCTTGCACTGTGGT	GCGCAAGCTTAATGCCCA-<br>AAACACAAAAATA
h-TTP-3'UTR-Mut	CCAAAGCCGTTAGACAACCCCACCCAT	ATGGGTGGGGTTGTCTAACGGCTTTGG
PHAGE-TTP	CGCGTCGACATGGATCTG-<br>ACTGCCATCTACGAG	GCGCTCGAGTCACTCAGAAA-<br>CAGAGATGCGATTG
m-siDrosha	AGAUCACCGUCUCUAGAAA	
m-siDicer	AGUGAGGUUUCAACGGAUCTT	
h-siDrosha	AACGAGUAGGCUUCGUGACUU	
h-siDicer	UGCUUGAAGCAGCUCUGGA	

siCtrl	TTCTCCGAACGTGTCACGTdTdT
h-miR-182-5p mimc	UUUGGCAAUGGUAGAACUCACACU
mimc Ctrl	UUUGUACUACACAAAAGUACUG
h-miR-182-5p inhibitor	AGUGUGAGUUCUACCAUUGCCAAA
inhibitor Ctrl	CAGUACUUUUGUGUAGUACAAA
